# Threshold for detection of diabetic peripheral sensory neuropathy using a range of research grade monofilaments in persons with Type 2 diabetes mellitus

**DOI:** 10.1186/1757-1146-1-9

**Published:** 2008-09-11

**Authors:** Mary P Thomson, Julia Potter, Paul M Finch, Richard B Paisey

**Affiliations:** 1 Department of Podiatry and Foot Health, Torbay Hospital, Devon, UK; 2 School of Health Professions and Rehabilitation Sciences, University of Southampton, Southampton, UK; 3 Chair of Health Sciences, Conestoga College, Ontario, Canada; 4 Diabetes Centre, Torbay Hospital, Devon, UK

## Abstract

**Aims:**

To identify the threshold of reduced sensory perception in Type 2 diabetes mellitus (Type 2 DM) using a range of research grade monofilaments.

**Methods:**

Three groups of participants were recruited into a between subject, cross-sectional study. Group 1(NEW), persons with Type 2 DM diagnosed for less than 2 years (*n *= 80); Group 2 (EST) persons with Type 2 DM diagnosed for more than 2 years (*n *= 91), and Group 3, a Comparison group without Type 2 DM (*n *= 73), resulted in a total study population, *n *= 244. Research grade monofilaments (2, 4, 6, 8 and 10-gram) were employed using standardised protocol, at 6 sites on the plantar aspect of both feet. The demographic and anthropometric measures of gender, age, height, weight, body mass index (BMI), blood pressure and duration of Type 2 DM since diagnosis (if applicable) of the participants were analysed.

**Results:**

Perception of the research grade monofilaments differed significantly between the 3 groups (p < 0.05). The 6-gram monofilament was found to be the threshold of normal perception, based on 90% of the Comparison group perceiving the 6-gram monofilament at all sites in contrast to 64% of NEW and 48% of EST groups.

**Conclusion:**

The 6-gram monofilament was identified as the threshold of normal sensory perception. Inability to perceive the 6-gram monofilament indicates, when using the method described in this study, that diminution of sensory perception is evident. Employing a range of monofilaments, 6, 8 and 10-grams in Type 2 DM foot screening would allow the clinical detection of deteriorating sensory perception and enable implementation of foot protection strategies at an earlier stage than is currently practised.

## Background

One of the common long-term complications of diabetes is neuropathy [1] of which there are several types with varying clinical features. The clinical definition of diabetic peripheral sensory neuropathy (DPSN) used in this study is:

'*a condition in which patients with diabetes experience symptoms such as pain, burning, hyperaesthesia, or are found to have signs of nerve damage of which they are unaware, principally the anaesthetic and deformed foot*.' [2]

It should be noted, however, that a number of persons with diabetes are aware of the signs of developing nerve damage in their feet.

Prevalence of DPSN varies widely in studies, depending on the criteria for diagnosis and the sensitivity of its detection [3]. A community-based study [4] found a prevalence of 42% in persons diagnosed with Type 2 diabetes for more than 2 years, of whom only 48% reported significant neuropathic symptoms.

It has been recorded that 99% of diabetes care is self-care [5], and it is part of the role of the health care professional to enable people to improve their diabetes-related coping skills, encouraging them to improve self-care behaviour, metabolic outcomes and thereby quality of life. The use of simple clinical tests allows a person with diabetes to understand a developing complication, such as loss of protective sensation in its early stages. This gives the knowledge and therefore the option to those with early Type 2 diabetes to make the behavioural changes that may be required to prevent foot problems such as ulceration and amputation.

The clinical design of this study was intended to produce results that could be easily interpreted by both clinician and layperson.

Whilst acknowledging that assessment of both vibration perception threshold and 10-gram monofilament together provide the most specific and most sensitive method of assessing sensory status in the diabetic foot [6], for the purpose of this paper the authors have concentrated on monofilament assessment alone [7-9].

The inability to perceive the 10-gram monofilament at non-callused sites on the plantar aspect of the foot indicates that there is a loss of protective sensation which increases the risk of neuropathic foot ulceration [7,9,10]. One study [11] calculated that lack of perception of the 10-gram monofilament equated to a 10-fold increased risk of ulceration. The employment of a range of monofilaments have been used since the development of the Semmes-Weinstein monofilaments circa 1950 [12] and there has been some disparity amongst researchers as to the minimum weighted monofilament that qualifies as the 'normal' threshold [13-15]. One study [16] concluded that perception of a monofilament of approximately 2-grams placed a person within the normal range whereas another [6] used a 5-gram monofilament as the reference for normal perception in the foot.

Therefore for this study a range of research grade monofilaments (2, 4, 6, 8 and 10-grams) applied to specific sites, avoiding overlying callus, were employed in order to identify the threshold for normal perception, thus enabling both clinician and person with diabetes to appreciate diminished sensory perception prior to the 10-gram threshold which indicates loss of sensory perception [7,9-11]. Earlier recognition of developing neuropathy by both person with diabetes and clinician allows discussion of behaviour changes necessary to protect the feet. This would include the introduction of daily inspection of feet and discussion relating to the appropriateness and necessity of having correctly fitting footwear, introduced to the person with diabetes over a period of time, rather than at a later stage when neuropathy is overt, when the clinician might well insist on these behaviour changes immediately, which might meet with resistance from the person with diabetes.

Three groups of participants were recruited; newly-diagnosed (less than 2 years since diagnosis); established (more than 2 years since diagnosis) and a comparison group without Type 2 diabetes mellitus or family history of it. It is recognised that the newly-diagnosed group may well have been developing Type 2 DM for some time prior to official diagnosis as would have been the case for the established group. However the intention was to investigate two separate cohorts of persons with Type 2 DM in order to enable comparison of those with Type 2 DM of longer duration with those of shorter duration as will be seen in the results section.

The sites tested by monofilament and number of applications at each site, vary widely in studies as may be seen in Table 1[17].

**Table 1:** The number of sites tested with 10-gram monofilament in various studies.

**Author (s)**	Number of Sites tested/recommended	No. of sites insensitive to represent PN
Holewski, Stess et al (1988)	6	3/6
Kumar, Fernando et al (1991)	1	1
Rith-Najarian, Stolusky et al (1992)	8	1 but retested twice
McNeely, Boyko et al (1995)	9	1/9
Dorgan, Birke et al (1995)	5	Not stated
Litzelman, Marriott et al (1997)	3	1
Frykberg, Lavery et al (1998)	3	2/3
Boyko, Ahroni et al (1999)	9	1/9
International Working Group on the Diabetic Foot (1999)	3	2/3 retested twice
Pham, Armstrong et al (2000)	1	1

PN = peripheral neuropathy

The six sites selected for testing for this study were the plantar aspect of hallux, first, second, third, fourth and fifth metatarsal heads, avoiding callus [18] with loss of perception to one site indicating loss of perception to that weight of monofilament [7,9,19,20].

Investigating specific sites in the forefoot with a range of monofilaments in order to ascertain when perception deviates from the expected threshold would allow both preventative therapeutic action and education to be initiated at an earlier stage than is currently the norm. This study was designed to identify the threshold at which it is clinically feasible to detect reduced sensory perception in Type 2 DM. Therefore the experimental hypothesis was: there is a difference between three groups, new and established Type 2 diabetic participants and a comparison group, and their perception of a range of research grade monofilaments applied to six sites on the plantar aspect of the foot.

## Methods

Ethical approval was granted by Torbay Local Research Ethics Committee. Information sheets were provided and written consent obtained from each participant prior to the commencement of the study. Persons with any condition (other than diabetes) associated with peripheral neuropathy or impaired nerve responses, cancer therapy (current or in the past five years), myocardial infarction, angioplasty or bypass graft, rheumatoid arthritis, alcoholism, history of or current ulceration, gross pedal deformity, use of walking aid and pregnancy were excluded from the study.

Three groups of participants were recruited for the study by convenience sampling and all were Caucasian except one South Asian. As indicated in Table 2, there was a group of 80 persons with Type 2 DM diagnosed for less than two years (NEW), and a group of 91 persons with established Type 2 DM of more than 2 years since diagnosis (EST). Both groups with Type 2 DM were recruited from G.P practices in the community and were recruited as they became available providing they fulfilled the inclusion and exclusion criteria. The third group of 73 participants (Comparison) did not have Type 2 diabetes mellitus or any known family history of the disease, with normal glucose tolerance on 75 gram oral glucose tolerance test, were recruited from friends and hospital staff.

**Table 2:** Participant demographics.

		**NEW *n *= 80**	**EST *n *= 91**	**Comparison *n *= 73**	**p value**
**Gender**	**Male**	**44**	**66**	**38**	= 0.013*
	**Female**	**36**	**25**	**35**	
**Age**	**Mean**	**59.6**	**61.4**	52.6*	<0.001*
	**+/- SD**	8.4	8.4	10.6	
**Height**	**Mean**	**170.7**	**171.4**	**172.6**	**= 0.45**
	**+/- SD**	9.1	8.7	9.5	
**Weight**	**Mean**	**94.8**	**89.2**	76.6*	<0.001*
	**+/- SD**	17	16	1.6	
**BMI**	**Mean**	32.7**	30.5**	25.5*	<0.001*
	**+/- SD**	6.1	5.5	3.4	= 0.016**
**HbA**_1_**c**	**Mean**	**8**	**8.3**	5.6*	<0.001*
	**+/- SD**	1.4	1.6	0.5	
**Systolic**	**Mean**	**141**	**136**	129*	<0.05*
	**+/- SD**	17.2	18.3	17.2	
**Diastolic**	**Mean**	82.7**	76.9**	**79.5**	<0.001**
	**+/- SD**	7.9	9.5	11.8	
**Duration**	**Mean**	**12.5**	**106.3**	**N/A**	**N/A**
**months**	**+/- SD**	7.4	78.2		

Key: * denotes that the Comparison group was significantly different from diabetic groups

** denotes significant difference between 2 diabetic groups

N/A = not applicable

The demographic and anthropometric measures of gender, age, height, weight, body mass index (BMI), blood pressure and duration of Type 2 DM since diagnosis (if applicable) of the participants were recorded.

In order to test sensory perception, research grade monofilaments (Bailey Instruments, Manchester) 2, 4, 6, 8, and 10-gram were employed in accordance with the predetermined protocol. This was informed by a pilot study [20], and incorporated the approach suggested by Booth and Young [21], in which each monofilament was 'bounced' three times prior to the test and not used more than 100 times in a 24 hour period.

In this study, inability to perceive a monofilament at any of the sites on the foot was recorded as loss of perception to that weight of monofilament. The test was clearly explained to the participant, and a monofilament was demonstrated on the inside of the investigator's forearm and then repeated at the same site on the participant. They were asked to say 'yes' every time they perceived the monofilament on their foot. Six sites were marked and tested on each foot; the participant closed their eyes and was therefore unable to see which site was being tested [20]. The testing of each site with a single monofilament took approximately 2 seconds, and the ascending method of limits was employed, commencing with the 2-gram monofilament. The six sites; pulp of hallux, 1^st^, 2^nd^, 3^rd^, 4^th ^and 5^th ^MTPJs on each foot were tested, once with each monofilament, but the timing between each test was varied. One investigator conducted all the monofilament testing. Data from both feet of the participants were collected and is available but right foot only data were analysed in detail, as in other studies [22]. Data were analysed using SPSS Release 10 software. Comparison of gender was tested by Chi-square and demographic, anthropometric and biochemical data between the three groups were tested by one way analysis of variance (ANOVA). Monofilament data were deemed to be ordinal and statistical analysis was performed using the Jonckheere-Terpstra (J-T) test, a non-parametric test for several independent samples [23].

At completion of data collection all monofilaments were retested by the manufacturer and they were found to deform to their relevant weight.

The level of significance selected for the results was 0.05 for hypothesis testing.

## Results

Data were collected from the 244 participants, see Table 2.

There was a significant difference between the three groups for gender (p = 0.013). The Comparison group were specifically recruited for this study (38 males, 35 females) whereas the other two groups were recruited by convenience sampling. As expected the Comparison group show lower blood pressure (systolic and diastolic), lower body weight, BMI and HbA_1_c than the groups with diabetes (ANOVA with Bonferroni multiple comparisons p < 0.001). Interestingly the BMI, and diastolic blood pressures are significantly greater in the NEW group compared with the EST diabetes group (p = 0.016 and p < 0.001 respectively), suggesting that optimum control of Type 2 DM in the NEW group had not yet been achieved. The Comparison group was significantly younger than the two diabetes groups, p < 0.001 (mean ages: NEW = 59.6; EST = 61.4 and Comparison = 52.6), which was due to the recruitment process.

**Figure 1:**
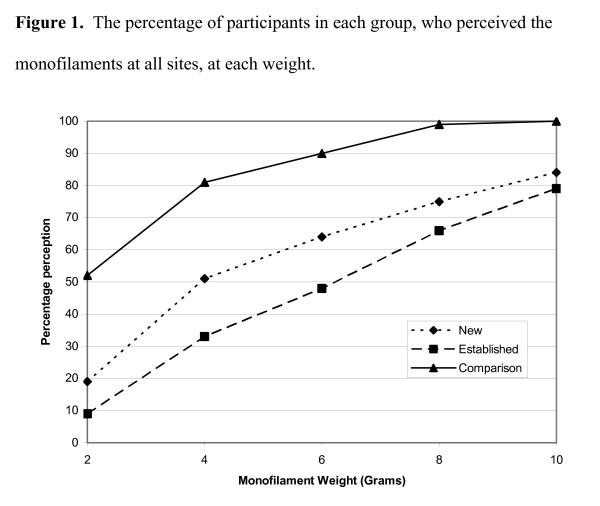


The results of the monofilament testing for the three groups at the six sites are presented in Figures 1, which shows the percentage of sites perceived in each group with each weight of monofilament on the right foot.

Figure 1 shows that only 19% of the NEW, 9% of the EST and 52% of the Comparison group, could perceive all six sites when the 2-gram monofilament was applied.

It can be seen that with the heavier 4-gram monofilament more participants in all 3 groups perceived it at all 6 sites; 51% of the NEW, 33% of the EST and 81% of the Comparison participants.

The numbers of participants in each group able to perceive the 6-gram monofilament at all sites increased again: 64% NEW; 48% EST and 90% Comparison.

When the 8-gram monofilament was applied, 75% NEW, 66% EST and 99% of the Comparison group perceived all six sites. And finally, 84% of NEW, 79% of EST and 100% of the Comparison group perceived the 10-gram monofilament at all sites.

The summary of monofilament perception at all sites and the Jonckheere-Terpstra test (2 degrees of freedom) results are shown in Table 3. This illustrates the results for the 6-gram monofilament, that is, 64% NEW, 48% EST and 90% of the Comparison group perceived the 6-gram monofilament at all sites, and puts them into the context of results from other weighted monofilaments. Figure 1 interprets the results in Table 3 graphically and demonstrates that the greatest difference between the 3 groups occurred with the results of the 4 and 6-gram monofilaments.

**Table 3:** The percentage of participants in each group, who perceived the monofilaments at all sites.

**Monofilament**	**NEW *n *= 80**	**EST *n *= 91**	**Comparison *n = 73***	**p value**
**2-gram**	19%	9%	52%	<0.001
**4-gram**	51%	33%	81%	<0.001
**6-gram**	64%	48%	90%	= 0.003
**8-gram**	75%	66%	99%	< 0.001
**10-gram**	84%	79%	100%	= 0.004

In the context of the results presented above, the null hypothesis was rejected as there were significant differences between the groups (p < 0.05).

## Discussion

In this study a series of research grade monofilaments; 2, 4, 6, 8 and 10-gram, were used at six sites on the plantar aspect of the foot to test sensory perception. The study showed that only 52% of the Comparison group could perceive the 2-gram monofilament at all sites, which suggests that its routine use for screening for diminished sensation is not appropriate. The results of the 4-gram monofilament suggest that a clinical sensory threshold was becoming apparent but only 81% of the Comparison group in our study could perceive the 4-gram at all sites and this percentage was considered too low to warrant recognition as the sensory threshold. However, two other studies, a Japanese study [24] and an American study [25] concluded that using the 4-gram and the 4.5-gram monofilament respectively, rather than the traditionally used 10-gram monofilament would be clinically useful for detecting DPSN at an earlier stage. When the 6-gram monofilament was employed in our study, the percentage of persons able to perceive it at all sites rose to 90% of the Comparison group, 64% of the NEW and 48% of the EST group. The 8-gram monofilament was perceived at all sites by 99% of the Comparison group and 75% of the NEW and 66% of EST respectively. These data indicate that while less than 100% of the Comparison group were able to perceive the 6-gram monofilament, it nevertheless showed a greater differential between the sensory capability of non-diabetic and diabetic participants than at 8-grams or 10-grams. The Comparison group were younger than the participants in the other two groups and this might have affected the results. In addition, the findings indicate that the group with diabetes of longer duration (EST) was less able to perceive the 6-gram monofilament than the group of participants with newly-diagnosed diabetes (NEW), reinforcing the notion that sensation diminishes as the duration of diabetes increases. It could be argued that 95% is most commonly used to indicate normality but this figure is probably achieved between 6 and 8-grams (unknown as a 7-gram monofilament was not used) after careful consideration the authors decided to use 90% as the level to indicate normality and therefore perception of the 6-gram monofilament as the threshold. Clinical judgement is required when interpreting results and false positives may be recorded when using the 6-gram monofilament, it should be clearly appreciated that loss of perception to the 6-gram monofilament is not diagnostic of DPSN. However, the main thrust of this study is to heighten the awareness of diminishing sensation prior to outright loss of protective sensation at 10-grams in order to empower the person with Type 2 diabetes to take ownership of their condition and enable them to take action in order to prevent problems developing in their feet.

The results suggest that using a range of monofilaments (6, 8 and 10-gram) provides the ability to detect diminished sensation at an earlier stage than use of the 10-gram monofilament alone. Persons with diabetes frequently do not regard DPSN as a problem unless it causes discomfort, and large discrepancies exist between foot care knowledge, perceived vulnerability and behaviour [26]. It might be anticipated that the use of a range of monofilaments could clearly demonstrate the need for increased vigilance with foot care to the person with diabetes. The employment of a range of monofilaments would allow protection and prevention programs for foot care to be introduced at an earlier stage as required by the National Service Framework for Diabetes [27].

The monofilaments used in this study are estimated, by the manufacturer Bailey Instruments, Manchester, using unpublished archive data on file (1998), to be able to endure approximately 24000 'bounces' before they lose their integrity (that is equivalent to 2000 patients using the testing protocol used in the study). Currently, the cost of an individual monofilament manufactured by Bailey Instruments Manchester is £14 plus VAT, therefore setting the cost of a series of monofilaments (6-gram, 8-gram and 10-gram) of £42 plus VAT against the mean weekly cost of £59 for a non-infected foot ulcer [28,29] clearly demonstrating the potential cost-effectiveness of a range of monofilaments.

The results of this study are based on the criteria of testing 6 sites and application of each monofilament once at each of those sites. Other workers have used different criteria; such as testing a different number of sites, from 1 site [7], 3 sites [11,19] to 9 sites [9] and applying the monofilament 3 times at each site and recording the majority response [11,30]. Another criterion in this study determined that the selected monofilament had to be perceived at all 6 sites whereas other studies used different criteria such as 3 sites perceived out of 6 [18]. Thus it may be seen that it is difficult to compare studies when different criteria are used. This problem could be resolved by the use of electrophysiological studies which are considered to be the gold standard for assessing and confirming neuropathy as the tests are sensitive, reliable and reproducible. However, the tests are limited as they are time-consuming, can be uncomfortable for the patients, and need skilled personnel to interpret the results [31]. The current study reveals that more research is required into the use of monofilaments. Future research should include the development of a standardised protocol incorporating the method of application to include selection and identification of the precise sites to be tested, number of applications at a particular site with each monofilament, the order of the sites tested by the individual monofilaments, and translation of results.

## Conclusion

Most (90%) healthy persons without Type 2 DM were able to perceive the 6-gram monofilament at the selected sites on the plantar aspect of the foot. It is therefore reasonable to use the 6-gram monofilament as a threshold measure for screening persons with Type 2 DM. Inability to perceive the 6-gram monofilament indicates, when using the method described in this study, that diminution of sensory perception is evident. The 8-gram monofilament should be employed if perception is not apparent at all sites and finally the 10-gram monofilament. This method would allow stepwise progression of education for the person with Type 2 DM at annual review.

## Abbreviations

ANOVA: analysis of variance; DM: diabetes mellitus; DPSN: diabetic peripheral sensory neuropathy; EST: participants diagnosed with Type 2 diabetes for more than 2 years; MTPJ: metatarso-phalangeal joint; NEW: participants diagnosed with Type 2 diabetes for less than 2 years.

## Competing interests

The authors declare that they have no competing interests.

## Authors' contributions

MT conceived and designed the study, undertook all data collection and analysis and wrote the paper. JP supervised the study at all levels from the design stages, interpretation of data through to completion of the paper to which she has given permission for publication. PF has been involved in intellectual discussions with regard to the study throughout, revising the manuscript for important intellectual content and has given approval for the final version to be published. RP has actively participated and supported the conception and design of the study, supervising all the recruitment of the participants actively discussed the interpretation and implications of results and proof read the final paper and given approval for the final version to be published.
